# Genomic Regions Associated with Tolerance to Freezing Stress and Snow Mold in Winter Wheat

**DOI:** 10.1534/g3.116.037622

**Published:** 2017-01-30

**Authors:** Erika B. Kruse, Scott W. Carle, Nuan Wen, Daniel Z. Skinner, Timothy D. Murray, Kimberly A. Garland-Campbell, Arron H. Carter

**Affiliations:** * Department of Crop and Soil Sciences, Washington State University, Pullman, Washington 99164; † United States Department of Agriculture-Agricultural Research Service, Wheat Health, Genetics and Quality Research, Washington State University, Pullman, Washington 99164; ‡ Department of Plant Pathology, Washington State University, Pullman, Washington 99164

**Keywords:** *Triticum aestivum*, QTL mapping, snow mold tolerance, freezing tolerance

## Abstract

Plants grown through the winter are subject to selective pressures that vary with each year’s unique conditions, necessitating tolerance of numerous abiotic and biotic stress factors. The objective of this study was to identify molecular markers in winter wheat (*Triticum aestivum* L.) associated with tolerance of two of these stresses, freezing temperatures and snow mold—a fungal disease complex active under snow cover. A population of 155 F_2:5_ recombinant inbred lines from a cross between soft white wheat cultivars “Finch” and “Eltan” was evaluated for snow mold tolerance in the field, and for freezing tolerance under controlled conditions. A total of 663 molecular markers was used to construct a genetic linkage map and identify marker-trait associations. One quantitative trait locus (QTL) associated with both freezing and snow mold tolerance was identified on chromosome 5A. A second, distinct, QTL associated with freezing tolerance also was found on 5A, and a third on 4B. A second QTL associated with snow mold tolerance was identified on chromosome 6B. The QTL on 5A associated with both traits was closely linked with the *Fr-A2* (Frost-Resistance A2) locus; its significant association with both traits may have resulted from pleiotropic effects, or from greater low temperature tolerance enabling the plants to better defend against snow mold pathogens. The QTL on 4B associated with freezing tolerance, and the QTL on 6B associated with snow mold tolerance have not been reported previously, and may be useful in the identification of sources of tolerance for these traits.

Winter wheat (*Triticum aestivum* L.) is planted in the autumn and harvested the following summer, to take advantage of winter moisture and increase yield (Schillinger and Young 2004). Millions of tons of grain yield are lost every year due to lack of winter-hardiness, which is the ability to survive the winter in a condition sufficient to thrive the following season. Under severe winter conditions, losses can be as high as 60–80% in winter wheat fields (Klein and Baenzinger 2015; Allan *et al.* 1992). Many factors contribute to winterkill, including freezing conditions and attack by pathogens active at low temperatures. Snow cover helps prevent freezing stress, but also facilitates snow mold infection by maintaining an environment suitable for the pathogens causing snow mold (Bruehl and Cunfer 1971). Multiple fungal species may be involved in the snow mold complex at a given location. Four different snow mold diseases, caused by seven soil-borne fungal or fungal-like species, occur in the US state of Washington: pink snow mold (caused by *Microdochium* [*Fusarium*] *nivale*), speckled snow mold (*Typhula idahoensis*, *T. ishikariensis*, and *T. incarnata*), snow scald (*Myriosclerotinia borealis*), and snow rot (*Pythium iwayami* and *P. okanoganense*) (Murray *et al.* 1999). Identifying sources of tolerance to this complex has been difficult. An early study of snow mold tolerance in the US Pacific Northwest examined >12,000 wheat accessions from around the world, but identified only 10 accessions with observable tolerance (Bruehl 1982). This work ultimately led to the development of the cultivar, Sprague, which has a useful level of snow mold tolerance (Bruehl 1982). Multiple loci appeared to confer this tolerance when its heritability was examined (Bruehl 1982). Iriki and Kuwabara (1993) found multiple genes involved in conditioning tolerance to other snow mold fungi. However, no sources of tolerance have yet been mapped to the wheat genome.

Cold-acclimation of winter wheat plants can result in an accompanying increase in snow mold tolerance (Gaudet and Chen 1987; Yoshida *et al.* 1998; Gaudet *et al.* 1999; Nishio *et al.* 2008); however, snow mold tolerance has also been found to occur independently of cold acclimation (Ergon and Tronsmo 2006). Although both freezing tolerance and snow mold tolerance have been associated with cellular carbohydrate dynamics during low temperature growth (Yoshida *et al.* 1998; Gaudet *et al.* 1999; Sugiyama and Shimazaki 2007), demonstration of a causative relationship has remained elusive.

From a gene expression perspective, it has been shown that cold acclimation is associated with the significant upregulation or downregulation of hundreds to thousands of wheat genes (Gulick *et al.* 2005; Monroy *et al.* 2007; Laudencia-Chingcuanco *et al.* 2011; Ganeshan *et al.* 2011; Winfield *et al.* 2009, 2010). Additional genes respond when acclimated plants are exposed to freezing temperatures (Herman *et al.* 2006; Skinner 2009, 2015), leading to greater tolerance of subsequent, potentially damaging freezing events (Herman *et al.* 2006; Skinner and Bellinger 2010). Many of the upregulated genes in these studies were identified as defense-related. Additionally, Gaudet *et al.* (2011) found that many of these defense-related genes were more strongly upregulated in snow mold tolerant lines than in susceptible lines during growth at low temperatures, even in the absence of the fungal pathogens. Christova *et al.* (2005) demonstrated that the protein product of a cold-inducible cystatin gene from wheat significantly inhibited hyphal growth of *M. nivale* in culture, implying that the products of certain wheat genes may incidentally create an environment inhospitable to some snow mold fungi. Taken together, these lines of evidence suggest that some combinations of genes promote freezing tolerance, other combinations promote snow mold tolerance, and other combinations promote both.

Selection for freezing and snow mold tolerance would be facilitated by the identification of molecular markers associated with genes conferring tolerance to these two afflictions, singly and in combination. The objective of this study was to identify such molecular markers.

## Materials and Methods

A population of 155 recombinant inbred lines (RILs) was derived from the F_2:5_ generation of a cross between two soft white winter wheat cultivars Finch (PI628640) and Eltan (PI536994). Eltan has moderate tolerance to both freeze damage and snow mold infection (Peterson *et al.* 1991), but Finch is susceptible to both freeze damage and snow mold infection (Garland-Campbell *et al.* 2005). Snow mold tolerance was evaluated on a scale of 0 (completely dead, with abundant mold) to 10 (thriving, with no mold) in 2013 (Waterville) and 2015 (Waterville and Mansfield) in field trials located in Douglas County, Washington. Freezing tolerance was assayed under controlled conditions via a modified protocol from Zhu *et al.* (2014). Of the 155 RILs, 149 were successfully evaluated for tolerance to both stressors and genotyped using the 9k iSelect SNP chip (Cavanagh *et al.* 2013) and 88 microsatellite markers (SSRs), as well as 20 90k iSelect SNPs of interest (Wang *et al.* 2014).

Twenty-one linkage groups were constructed in Joinmap 4 (Van Ooijen 2011) using maximum likelihood mapping from 662 nonsynonymous markers. Phenotyping data were transformed using a beta binomial distribution with a logit link function to correct for skewing. Normality of both the snow mold and freezing tolerance data were improved via this transformation. Best linear unbiased predictors (BLUPs) were created for each trait using the procedure for generalized linear mixed models (Proc GLIMMIX) of SAS 9.3 (SAS Institute, Cary, NC). QTL analysis was performed using composite interval mapping in QTL Cartographer Version 2.5 (Basten *et al.* 2004), and the linkage groups were graphed using MapChart 2.1 (Voorrips 2002). Additionally, in order to confirm the identity of the primary discovered QTL, the RILs were genotyped with the KASP marker S2269949, associated with *Fr-A2* (Sieber *et al.* 2016) and mapped.

### Data availability

Additional data and materials can be made available upon request. A full description of the protocol used is available in Supplemental Material, File S1. All marker calls, linkage map information, marker names, raw phenotype data, processed phenotype data, code and fit statistics are present in File S2. A table containing the full names and positions of all markers on the linkage groups where significant QTL were found is present in the Table S1.

## Results

### Evaluation of snow mold and freezing tolerance

**Figure 1:**
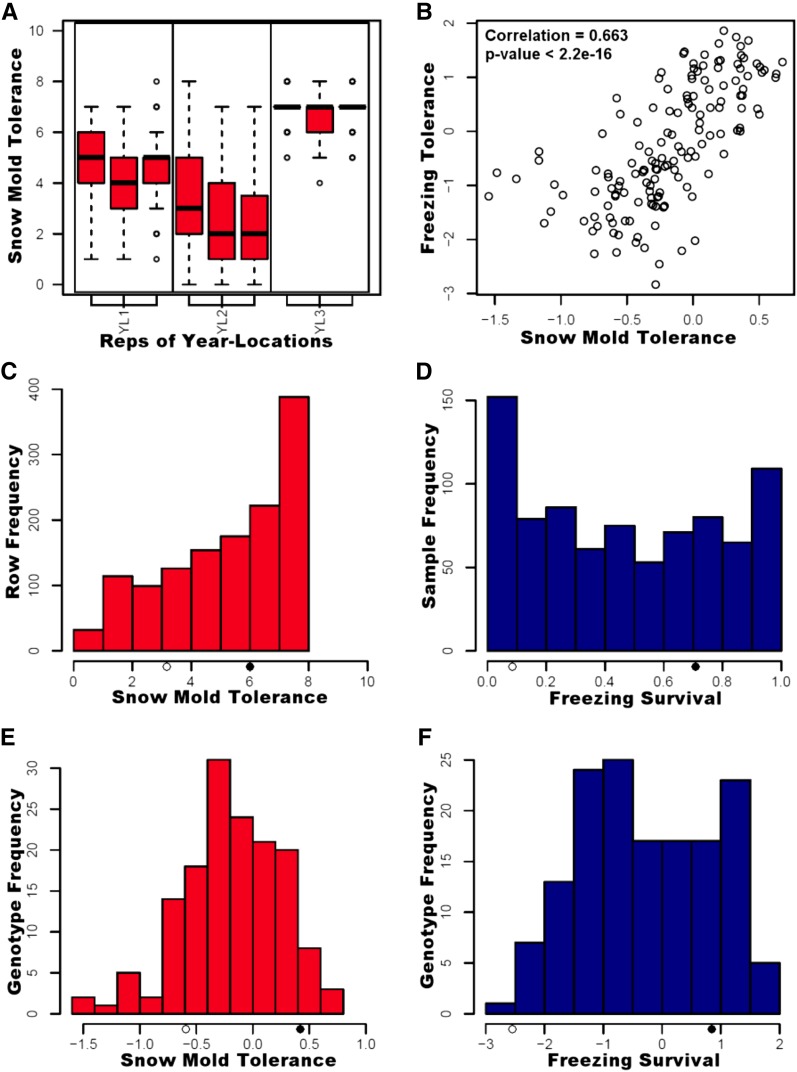
(A) Boxplots of snow mold tolerance data showing distributions of the ratings in each replicate of each year-location (YL). (B) Correlation between freezing BLUPs and snow mold BLUPs. (C) Histogram of all raw snow mold tolerance data for each year-location. (D) Histogram of raw freezing test survival rates with replication. (E) Histogram of BLUPs for snow mold year-locations. (F) Histogram of BLUPs for freezing tolerance data. (C–F) Average phenotypes of the susceptible and tolerant parental lines are represented by open and closed circles respectively on the *x*-axis.

Finch was less tolerant to snow mold infection than Eltan, and segregation for snow mold tolerance was observed among the RILs. The distributions of ratings for each snow mold trial are shown in Figure 1A.

As was expected, there was a positive correlation between the freezing tolerance and snow mold tolerance scores; however, there were also unique features in the data (Figure 1B) indicating unique mechanisms governing the traits. The freezing survival approximated a binomial distribution, but was also zero inflated, whereas the snow mold data distribution was highly skewed (Figure 1, C and D). Tolerance of some lines fell outside the parental range, and may be transgressive segregants (Figure 1, C–F).

**Figure 2:**
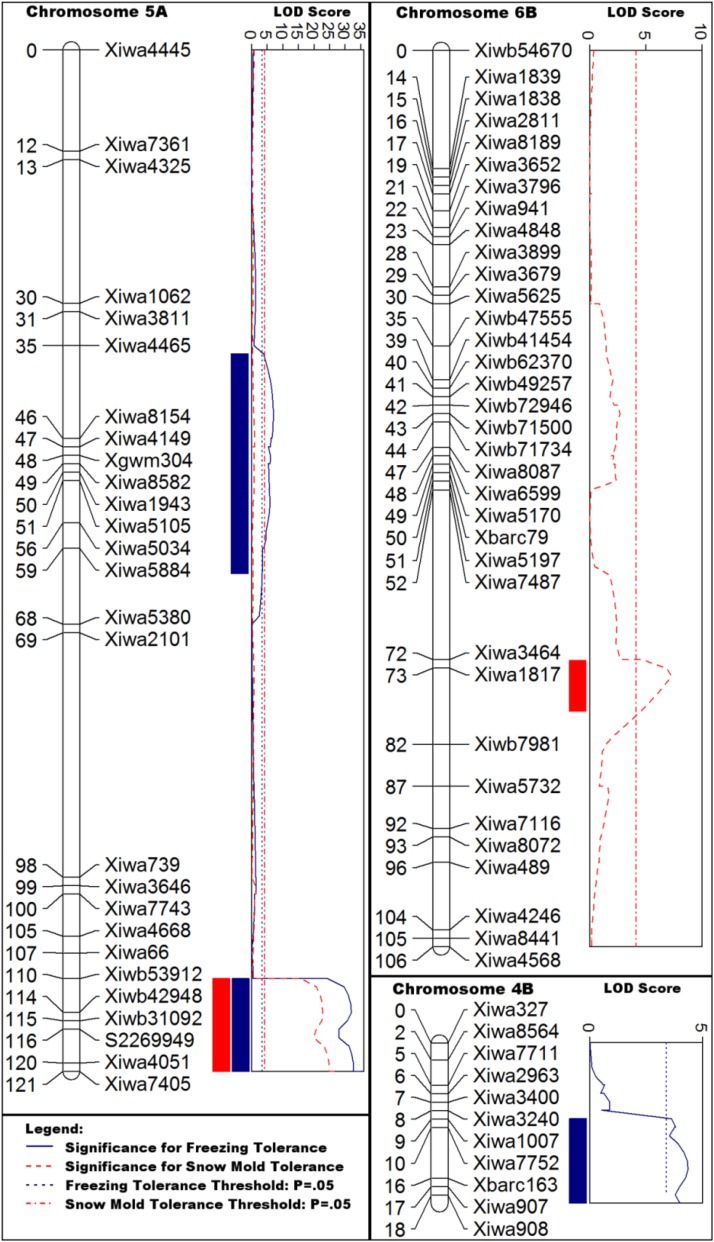
Detected QTL: Displayed marker density has been reduced to 1 cM on these graphs, but all relevant marker data are available in Table S1. Blue bars, significant markers for freezing tolerance. Red bars, significant markers for snow mold tolerance.

For both freezing tolerance and snow mold tolerance, the QTL detected on the distal end of the linkage group on chromosome 5A had the highest significance and the largest effect (Figure 2 and Table 1). Significance of this QTL was detected in each snow mold year-location singly and in combination. The tolerant parental genotype, Eltan, conveyed this source of tolerance, and LODs for QTL ranged from 26 to 35. Localization of the QTL corresponds with *Fr-A2*, which was confirmed with the known KASP marker S2269949 (Sieber *et al.* 2016) that mapped between markers *Xiwb42948* and *Xiwa7405* on the distal end of the 5A linkage group. Chromosome 5A continues beyond the distal end of this linkage group, but of the 129 SNP markers that colocalize with that region that were genotyped for this study, all were monomorphic between the parental genotypes.

**Table 1:** Flanking markers, positions, and significance of each of the discovered QTL

QTL	Trait	Chr	Markers	Position Range (cM)	Max LOD*^a^*	Threshold	Max R2 (%)*^b^*	Source
QFSelt.wpg-5A.l	SMT	5A	*Xiwb53912-Xiwa7405*	110.101–121.46	26.37	4.1	47	Eltan
Fr-A2	FRT	5A	*Xiwb53912-Xiwa7405*	110.101–121.26	34.60	3.6	49	
QFelt.wpg-5A.2	FRT	5A	*Xiwa4465-Xiwa5380*	35.603–68.155	6.99	3.6	8	Eltan
QSfin.wpg-6B	SMT	6B	*Xiwa7487-Xiwb7981*	52.008–82.514	7.31	4.1	10	Finch
QFfin.wpg-4B	FRT	4B	*Xiwa3240-Xiwa908*	8.811–18.953	4.37	3.6	4	Finch

All markers associated with these QTL are listed in Table S1.

a The maximum LOD score for each QTL region is reported.

b The maximum R2 value for each QTL region is reported. This value may not occur at the exact position of the maximum LOD score.

For snow mold tolerance alone, a QTL was detected on the chromosome 6B linkage group (Figure 2 and Table 1). Insufficient statistical power prevented reliable detection of this QTL when the year-locations were examined individually; however, the combined BLUP analysis yielded a peak more significant than was present in the BLUPs from any individual year-location. The 6B QTL had a maximum LOD score of 7.31, and was conferred by the susceptible parent, Finch.

### Identification of QTL for snow mold and freezing stress tolerance

**Figure 3:**
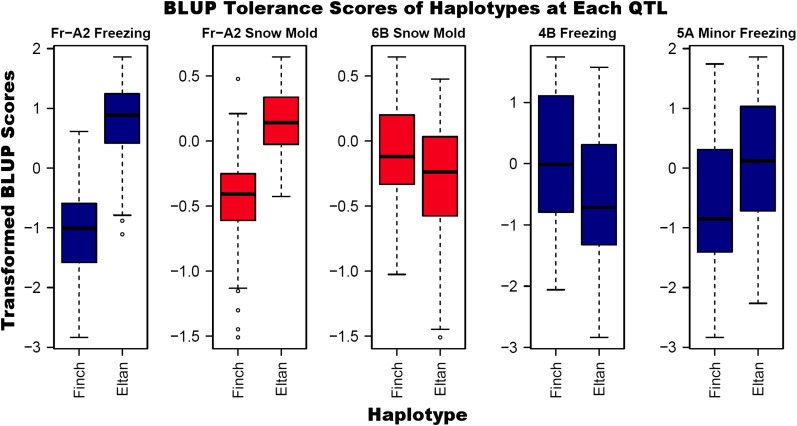
Boxplots contrasting the distributions (with quartiles and the median) of all genotypes with either the “Finch” or “Eltan” haplotype.

For freezing tolerance alone, a significant QTL was also detected on 4B, with a LOD score of 4.37. This source of tolerance was also conferred by the susceptible parent, Finch (Figure 3).

A second QTL for only freezing tolerance was detected on chromosome 5A and located between markers at 36–68 cM. The source of this tolerance came from Eltan (Figure 3).

## Discussion

The finding of multiple QTL associated with freezing tolerance on chromosome 5A is consistent with previously published reports. In fact, QTL have been found on various chromosomes throughout the genome, as is the case in this study. For instance, three studies of freezing tolerance identified QTL on chromosomes 1A, 1D, 2A, 2B, 3A, 5A, 5B, 6A, 6D, and 7B (Fowler *et al.* 2016; Båga *et al.* 2006; Case *et al.* 2014). Each of these studies examined different freezing stress conditions, but they demonstrate the quantitative nature of freezing tolerance. Upon comparison, the studies raise some interesting questions regarding the details of mapping on 5A. When the critical markers defining the QTL on chromosome 5A are compared using the 92k consensus linkage map (Wang *et al.* 2014), we find both overlapping and unique features between our findings and those previously reported. A unique feature of our map is the minor QTL for freezing tolerance found at 36–59 cM of chromosome 5A (Figure 2). Based on the consensus map, this locus has not been previously detected (Case *et al.* 2014; Fowler *et al.* 2016).

Our primary 5A locus was inseparable from both the Norstar × Manitou LT50 and the Cappelle Desprez × Norstar LT50 loci that are at ∼124 cM in the Fowler *et al.* (2016) linkage map. This same locus appears to overlap with the Case *et al.* (2014) locus that was identified as being close to *Fr-A2*. This locus is physically coincident with the *Fr-A2* locus—a region of chromosome 5A that has been investigated many times (Vagujfalvi *et al.* 2005; Motomura *et al.* 2013; Pearce *et al.* 2013; Todorovska *et al.* 2014; Zhu *et al.* 2014). Copy-number variation among the genes at the *Fr-A2* locus appears to impact freezing tolerance (Zhu *et al.* 2014; Sieber *et al.* 2016); however, the genomic arrangement of these copies is still unknown.

Occurrence of the Eltan allele at the *Fr-A2* locus improved snow mold tolerance in our study. The observation of improved snow mold tolerance associated with this QTL may result from a more effective fungal resistance mechanism(s) potentially due to pleiotropic effects. This is likely because the C-repeat binding factor (CBF) genes cause the upregulation of many cold-responsive genes, and snow mold tolerance mechanisms may also be subject to this upregulation (Skinner 2015; Miller *et al.* 2006; Fowler *et al.* 2005). Cold-responsive genes have been implicated for imparting not only freezing tolerance, but also snow mold tolerance (Christova *et al.* 2005; Gaudet *et al.* 2011). Alternatively, reduced freezing damage in the field may result in healthier plants that are more capable of combatting fungal infection. Future snow mold tolerance studies should choose parents with similar freezing tolerance. This would improve the statistical power of identifying new QTL for snow mold tolerance.

The detection of a QTL on chromosome 6B associated only with snow mold tolerance is unique to this study. In winter Triticale (*Triticosecale* X), QTL associated with components of tolerance to *M. nivale* have been detected on chromosomes 1B, 2A, 3A, 3B, 5A, 5B, 6A, 6B, and 7B (Szechyńska-Hebda *et al.* 2011). A review of the literature suggests that this 6B QTL is the first published locus in wheat to convey tolerance to the snow mold disease complex. Its identification in the US Pacific Northwest means that, while it is suited to the disease complex found there, it should be tested in other regions where wheat is afflicted by snow mold. The causal genes are unknown, but, upon publication of the wheat genome, candidates may be identified. Those insights may apply to other species that suffer from this snow mold infections, in addition to wheat.

The freezing tolerance QTL found on chromosome 4B has not previously been mapped. However, chromosome 4B has been implicated in early studies of freezing tolerance using chromosome substitution or deletion lines (Law and Jenkins 1970; Veisz and Sutka 1989; Sutka 1981, 2001). Like the snow mold tolerance QTL on chromosome 6B, the underlying genes are not yet known. Both 4B and 6B tolerance QTL are contributed by Finch, the susceptible parent, which means Eltan, the tolerant parent, did not possess some useful genetics conferring tolerance to these stresses. The combination of tolerance alleles from each of these parents is concurrent with the observation of possible transgressive segregants in this population (Figure 1, C–F). Thus, breeders may be able to implement unexpected sources of tolerance to recognize further gains in existing tolerant genotypes.

We have identified several novel QTL with minor effects; two for freezing tolerance on 4B and 5A, one for both freezing and snow mold tolerance on 5A, and one for only snow mold tolerance on 6B. These data provide useful information for wheat genetics, but also offer potential implications in related organisms. Additional work will be necessary to adapt these colocalizing SNPs into reliable markers, but these QTL will be useful in screening genotypes to create new cultivars with both freezing and snow mold tolerance.

## Supplementary Material

Supplemental material is available online at www.g3journal.org/lookup/suppl/doi:10.1534/g3.116.037622/-/DC1.
