# Supplementary figures and images for: Genomic Regions Associated with Tolerance to Freezing Stress and Snow Mold in Winter Wheat
Source: G3 (Bethesda). 2017 Jan 30;7(3):775–80. doi: 10.1534/g3.116.037622 (PMC5345707; doi:10.1534/g3.116.037622)

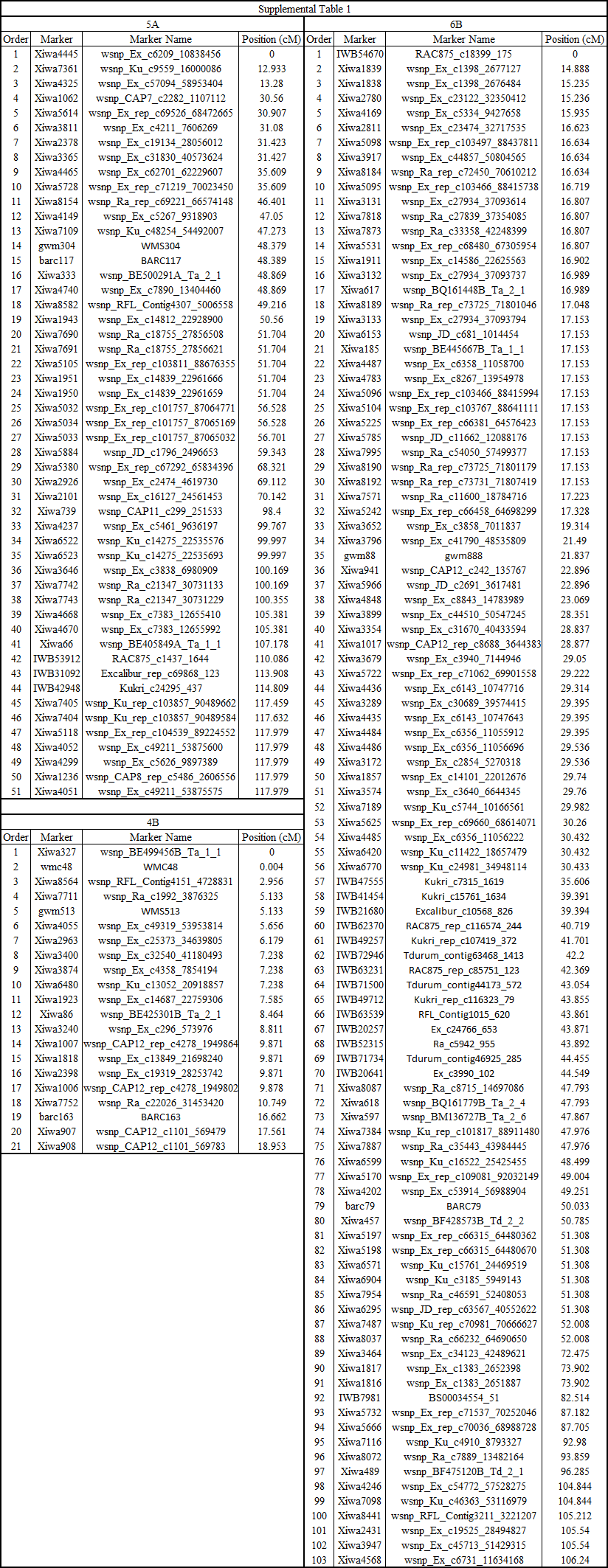

Supplement: Supplementary file 1 [file 775TableS1.tiff]
