# Supplementary material for: Genomic Regions Associated with Tolerance to Freezing Stress and Snow Mold in Winter Wheat
Source: G3 (Bethesda). 2017 Jan 30;7(3):775–80. doi: 10.1534/g3.116.037622 (PMC5345707; doi:10.1534/g3.116.037622)
Supplement: Supplementary file 2 [file 775FileS1.docx]

Supplemental File S1

Materials Methods Extended:

Plant Material

A population of 155 recombinant inbred lines (RILs) was derived from the F_2:5_ generation of a cross between soft white winter wheat varieties Finch and Eltan. Eltan (PI536994) has moderate tolerance to both freeze damage and snow mold infection (Peterson et al. 1991) and has been used as a source of winter-hardiness in the production of soft white winter wheat cultivars for the Pacific Northwest. Finch (PI 628640) is susceptible to both freeze damage and snow mold infections (Garland-Campbell et al. 2005). It has been used as a source of disease resistance with good yield potential and end-use quality. The cross yielded 167 F_2_ genotypes, from which 155 genotypes were advanced using single seed descent to the F_5_ generation, and then 149 were advanced as individual families and evaluated for tolerance to both stressors. Some genotypes failed to survive advancement.

Methods Used to Determine Snow Mold Tolerance

Snow mold tolerance was evaluated in 2013(Waterville) and 2015(Waterville and Mansfield) in field trials located in Douglas County, Washington. This region commonly has conditions favorable to snow mold, and growers rely on tolerant wheat varieties. The plots were planted into areas of natural infestation that have long histories of snow mold outbreaks.

For disease evaluation, the RILs were replicated three times as short rows in a randomized complete block design with the parents and the moderately tolerant cultivar 'Bruehl' repeated several times within each block. Both parental lines were replicated 11 times overall, and Bruehl was replicated 31 times. The trials were planted in the fall and managed by the collaborating grower’s preferred methods. Ratings were taken in the spring, when the snow had melted and the fields were accessible, at which point the plants were approaching stage 30 on the Zadoks (1974) growth scale. The plants were visually rated on a scale of 0 (completely dead, with abundant mold) to 10 (thriving, with no mold).

Methods Used to Determine Freezing Stress Tolerance

Twenty seeds from each genotype were planted in 48-well 30.5 cm X 61 cm trays in a completely randomized design. Multiple reps of the parental lines were used as internal controls and were planted in each of the trays (Zhu et al. 2014). The seeds were planted approximately 0.5 cm deep in Sunshine LT5 potting soil (Sungro Horticulture, USA) and were grown for 7 days at 22°C with 16-hour day-length before being transferred to 4°C with 12-hour day-length for 6 weeks in order to cold acclimate. Lighting conditions were kept at approximately 16,000 lm (200 µE) under LED growth lights. Plants were watered with nutrient water once during the first week and once every three weeks during their cold acclimation.

After acclimation, emergence of seedlings was counted for each genotype, and the flats were saturated with 10mg/L Snomax solution (Snomax LLC, Englewood, CO) and covered with approximately 2 cm of crushed ice in order to facilitate even freezing. The flats were placed in a LU-113 Low temperature cabinet (Espec Corp., Hudsonville, MI, USA) and brought through a freezing cycle of 16 hours at -3°C, 3.75 hours decreasing from -3°C to

-14°C, 1 hour at -14°C, and then gradually warmed to 4°C over the course of 4.5 hours. All temperature changes were at a constant rate of 4°C per hour. Plants were then removed from the chamber and kept at 4°C under lit conditions for 24 hours before being transferred to 22°C greenhouse conditions. Freezing tolerance was measured by counting the seedlings which were able to re-grow 4 weeks after the freezing stress treatment, and taking that number as a percentage of the total that emerged. Four replicates of the entire population were evaluated, and the average survival of those replicates was used for QTL mapping.

Marker Data and Linkage map construction

The parents were genotyped using the 9k iSelect SNP chip (Cavanagh et al. 2013) and 88 microsatellite markers (SSRs), as well as twenty 90k iSelect SNPs of interest (Wang et al. 2014) that were assayed with the Sequenom MassARRAY platform (Gabriel et al. 2009). Genome Studio v2011.1 software (Illumina, San Diego, CA, USA) was used to score the SNP markers and distinguish between those segregating in the population and those that were non-polymorphic. Of the initially identified 1259 polymorphic markers, synonymous markers were omitted from further analysis. Of the remaining 663 markers, none exhibited greater than 10% heterozygosity or greater than 63% prevalence of one haplotype. The 663 markers were used to define the genotypes of the recombinant inbred lines. Joinmap 4 (Van Ooijen. 2006) was used to assign the markers to 21 linkage groups (using a LOD threshold of 8) that cover 15 of the 21 wheat chromosomes. Distances within those linkage groups were subsequently determined with maximum likelihood mapping that employed five rounds of optimization, chain lengths of 1,000, and termination after 100,000 chains without improvement. The resulting map spans 1153 cM across its linkage groups.

The freezing tolerance data were zero-inflated and heavily right skewed. The snow mold data were also highly skewed, rather than normally distributed. Therefore, a beta binomial distribution was used with a logit link function to model the data, resulting in distributions with AIC values of -1884.29 for snow mold tolerance, and -1239.62 for freezing tolerance. The Pearson’s chi-square fit statistic failed to reject the null hypothesis for normality The analysis was performed using the GLIMMIX procedure of SAS 9.3 (SAS Institute, Cary NC). The best linear unbiased predictors (BLUPs) from these analyses representing the freezing tolerance and snow mold tolerance data of 149 recombinants were calculated.

QTL Analysis

QTL analysis was performed using QTL Cartographer Version 2.5 (Basten et al. 2004) and the BLUPs that had been transformed to the logit scale for the beta binomial analysis. Composite interval mapping was performed for each trait independently with a reading frame of 10 cM, a walk-speed of 1 cM, and 500 permutations. Visual representations of the QTL on respective linkage groups were generated using MapChart 2.1 (Voorrips, 2002). Confirmation of the identity of the primary QTL that was discovered was achieved through use of the KASP marker S2269949. This marker is associated with *Fr-A2* (Sieber et al., 2016), and was mapped into the primary QTL on the distal end of chromosome 5A.

**Supplemental Citations:**

Gabriel S., Ziaugra L., Tabbaa D., (2009). SNP genotyping using the Sequenom MassARRAY iPLEX platform. Current Protocols in Human Genetics, 2-12. doi: 10.1002/0471142905.hg0212s60

Zadoks J.C., Chang T.T., Konzak C.F., (1974), A decimal code for the growth stages of cereals. Weed Research 14: 415–421. doi:10.1111/j.1365-3180.1974.tb01084.x
